# School suspension predicts trichomoniasis five years later in a matched sample

**DOI:** 10.1186/s12889-020-8197-8

**Published:** 2020-01-20

**Authors:** Janet E. Rosenbaum

**Affiliations:** Department of Epidemiology and Biostatistics, School of Public Health, SUNY Downstate Health Sciences University, 450 Clarkson Ave, MS 43, Brooklyn, NY 11203 USA

**Keywords:** Propensity scores, Adolescent health, Emerging adulthood, Sexually transmitted diseases, Trichomoniasis

## Abstract

**Background:**

Young adults who were suspended from school during adolescence are more likely than matched non-suspended youth to be arrested, on probation, or not graduate from high school, which are STI risk factors. This study evaluates whether suspension is a marker for STI risk among young adults who avoid subsequent negative effects.

**Methods:**

This study evaluated whether suspension predicts a positive test for chlamydia, gonorrhea, or trichomoniasis in a urine sample using matched sampling in the National Longitudinal Study of Adolescent and Adult Health (Add Health), and evaluated potential mediators between suspension and STI status using causal mediation analysis. We used Mahalanobis and exact matched sampling within propensity score calipers to compare 381 youth suspended for the first time in a 1-year period with 980 non-suspended youth. The suspended and non-suspended youth were similar on 67 pre-suspension variables. We evaluated STI outcomes 5 years after suspension.

**Results:**

Before matching, suspended youth were more likely to test positive for trichomoniasis and gonorrhea, but not chlamydia, than non-suspended youth. Suspended youth were more likely to test positive for trichomoniasis 5 years after suspension than matched non-suspended youth (OR = 2.87 (1.40, 5.99)). Below-median household income before suspension explained 9% of the suspension-trichomoniasis association (*p* = 0.02), but criminal justice involvement and educational attainment were not statistically significantly mediators.

**Conclusions:**

School suspension is a marker for STI risk. Punishing adolescents for initial deviance may cause them to associate with riskier sexual networks even if they graduate high school and avoid criminal justice system involvement. Suspension may compound disadvantages for youth from below-median-income families, who have fewer resources for recovering from setbacks.

## Summary

Adolescents who were suspended from school for the first time had greater trichomoniasis risk than non-suspended adolescents propensity-matched on 67 covariates, and the association was mediated by below-median family income.

## Background

The persistent racial and socioeconomic disparities of sexually transmitted infections (STIs) raise questions of which racially disparate social structures contribute to these disparities. Racial disparities in STIs seem to be related to network effects, rather than riskier individual sexual behavior [1]. Education has been recognized as a social determinant of health [2], and school suspension appears to be the largest factor in the black-white gap in high school graduation [3]. However, it’s unknown whether school suspension predicts STIs. This study evaluates whether youth who are suspended from school for the first time are more likely to test positive for chlamydia, trichomoniasis, and gonorrhea 5 years after first suspension than similar youth who had never been suspended, and uses causal mediation to evaluate the roles of household income during adolescence. This study uses tests for chlamydia, trichomoniasis, and gonorrhea as objectively assessed potential health effects of suspension to avoid potential differential reporting, as would be the case for a self-reported health effect.

Implemented during the peak of school violence in the mid-1990s, the federal Gun-Free Schools Act mandated school suspension for weapons and illegal drugs; subsequently, states and localities mandated school suspension for non-violent and subjective offenses [4]. The most recent nationally representative estimates of the lifetime incidence of school suspension over grades K-12 are 44% for males (67% of Black males) and 25% of females (44% of Black females) [5]. Black youth appear to be suspended more than non-Black youth due to racial discrimination: psychology experiments with vignettes have found that educators are more likely to suspend Black youth than White youth [6].

Adolescents often experiment with risk behavior, but the theory of labeling suggests that punishing this experimentation with school suspension may cause youth to be labeled, stigmatized, join risky social networks, and subsequently engage in riskier behavior [7–9], as have been found in studies of police stops and arrests [10, 11]. Suspended youth may also engage in deviance amplification with both health and non-health behaviors: suspended youth are more likely to smoke tobacco [12], use marijuana [13], and engage in antisocial behavior [14] in the year after suspension than youth who were not suspended. However, these results may be explained by selection bias: suspended youth may have engaged in risk behavior even if they were not suspended. One study found no difference in educational attainment two years after suspension and inferred that previous studies could be explained by selection bias [15]. However, a longitudinal study that used matched sampling found suspended youth have lower educational attainment and greater criminal justice involvement than matched non-suspended youth 12 years after school suspension [16].

Education, race, and economic disadvantage are all associated with biomarker-detected STIs. Four-year college degrees (versus some or no college) are protective among women [17]; two-year college degrees (versus current enrolment in two-year college) are protective among young adults [18]; and men in the National Job Training Program have below-average educational attainment and greater chlamydia prevalence than men in nationally representative samples [19]. However, racial disparities are more pronounced than educational disparities; Black female college graduates have higher assay-determined STI risk than White females without a high school diploma [20]. Economic disadvantage is also a risk factor for STIs. Adolescents’ household income quintile predicts their risk of STIs 6 years later, at ages 18–25 [21]. This study uses nearest-neighbor Mahalanobis and exact matching within propensity score calipers to evaluate whether youth who were suspended for the first time during a one-year period were more likely to test positive for STIs 5 years later than matched non-suspended youth not suspended during that period. This study is novel for using a statistical matching method to minimize potential confounding and selection bias, and causal mediation analysis to evaluate potential pathways between suspension and STIs.

## Methods

### Data

We evaluate the association between school suspension and STIs using the National Longitudinal Study of Adolescent and Adult Health (Add Health), which remains the only nationally representative longitudinal study in the United States with STI test results. Add Health comprises a nationally representative sample of adolescents attending public and private high schools and their feeder middle schools in 1994–95 who were interviewed in their homes in 1995 (wave 1, response rate 79.0%), 1996 (wave 2, response rate 88.6%), and 2001 (wave 3, response rate 77.4%). We also used data from interviews with participants’ parents (93% female parents) in 1995 (response rate 82.5%) and school administrators in 1995 (response rate 97.7%.) [22]

**Fig. 1:**
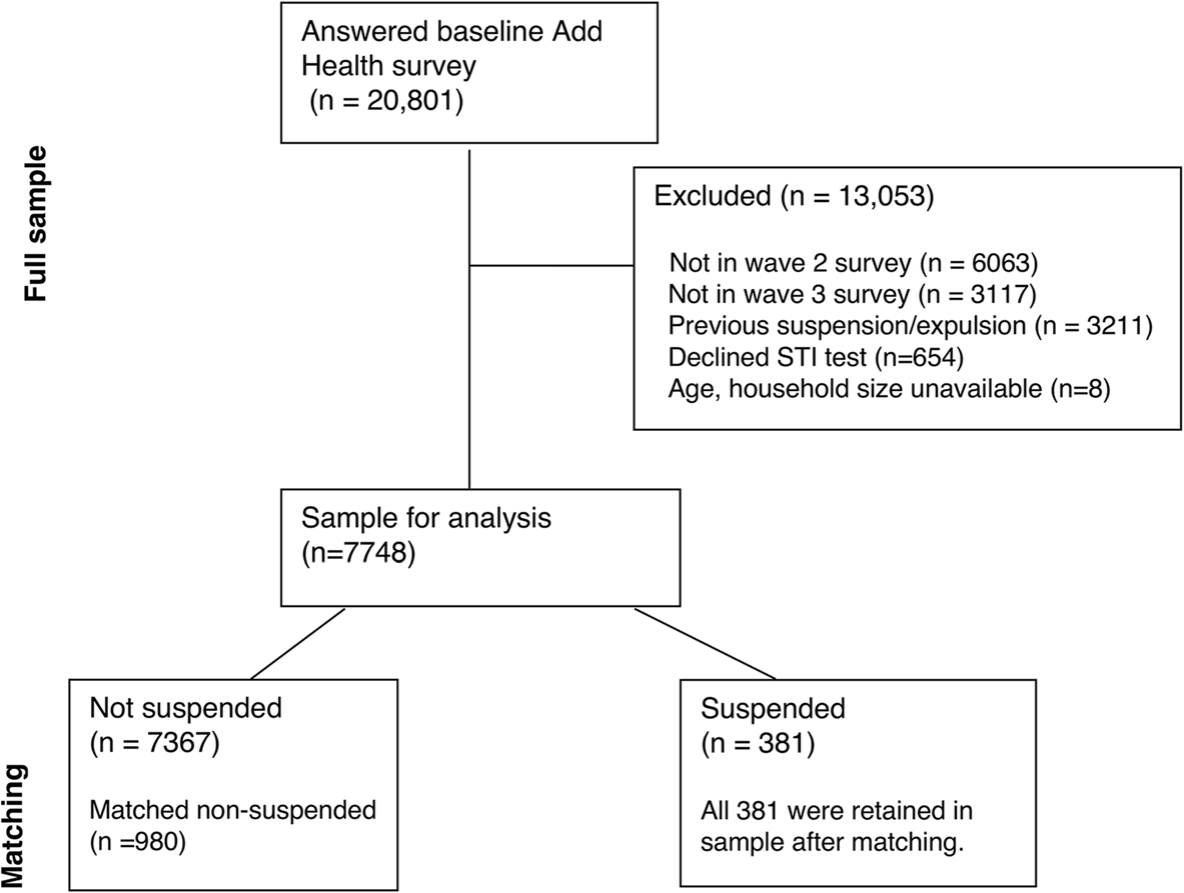
Construction of matched sample

We used data from 7748 respondents who participated in waves 1, 2, and 3 who reported that they had never received an out-of-school suspension (“Have you ever received an out-of-school suspension from school?”) or been expelled from school (“Have you ever been expelled from school?”); reported their birthdate and household size at baseline; and gave a urine sample for the STI tests. Respondents who gave a urine sample for STI testing received an extra $10 incentive; 91.5% of unmarried high school graduates gave a sample. Fig. 1 shows the construction of the sample and matched sample. We did not use the survey weights, because they were developed for the entire sample; developing new weights for a highly constrained sub-sample could induce bias [23].

### Predictor: new suspension in 1995–96

We measured a first school suspension between 1995 and 1996 from an affirmative answer to the wave 2 question, “During this school year (during the 1995–96 school year) did you receive an out-of-school suspension from school?” Because the sample was limited to participants without a prior out-of-school suspension or expulsion (see previous section), a suspension reported at wave 2 represents is a first lifetime suspension.

### Outcomes: positive STI tests

Three STI outcomes were measured in 2001, 5 years after suspension: testing positive for Chlamydia trachomatis (chlamydia), *Neisseria gonorrhoeae* (gonorrhea), or Trichomonas vaginalis (trichomoniasis). STI tests that did not return results (357 chlamydia, 810 gonorrhea, and 413 trichomoniasis) were coded as missing, so the sample sizes are slightly different for each STI.

Chlamydia and gonorrhea screening used Ligase Chain Reaction amplification technology in the Abbot LCx Probe System. Trichomoniasis was detected with a PCR-ELISA test for Trichomonas vaginalis DNA which has a sensitivity of 91% in women compared with combined reference standard of wet mount and culture from vaginal swab and 89% in men compared with urethral swab culture, and an adjusted specificity of 93% in women and 95% in men [24]. Chlamydia and gonorrhea tests were FDA-approved, but the trichomoniasis test was not yet FDA-approved, so only chlamydia and gonorrhea test results were made available to participants.

### Control variables

We identified 67 potential confounders of the relationship between suspension and STIs using Gottfredson and Hirschi’s self-control theory of deviance (3 s) and from past research about suspension [5, 25], educational attainment [26], and arrest [27], including demographics, socioeconomic status, sexual risk-taking, relationships with adults, educational factors, parents’ risk behavior, substance use, personality and mental health, and deviance. The control variables are listed in full in Appendix.

The control variables were measured at baseline, except for father ever in prison, which was measured in 2001. Father-in-prison measurement was used as a control variable because it was not likely to be a consequence of their child’s school suspension. The father could have gone to prison after the child’s school suspension, but the father’s propensity to go to prison likely existed prior to the child’s school suspension.

### Negative control

Randomized laboratory experiments routinely include a negative control: a condition under which a null result is expected; if a negative control condition does not produce a null result, that suggests a problem with the experiment. The propensity matching approach used in this paper mimics a randomized experiment; in this case, we use a negative control to detect residual confounding after matching [28]. We used post-suspension impulsivity, measured in 2001, as a negative control because we do not expect post-suspension impulsivity to be greater in suspended than matched non-suspended youth. Impulsivity was the sum of nine Likert-type scale items on a scale from 0 to 1 (α = .94). After matching, suspended and non-suspended youth did not differ on baseline constructs in the Gottfredson-Hirschi self-control model, including systematic versus gut-feeling decision-making [29] The same 9-item impulsivity scale was not available at baseline, so it could not be used for matching.

### Data analysis

We conducted analyses in the R statistical package 3.5.1.

### Bivariate analysis

We identified variables that differed between suspended and non-suspended youth using standardized differences, a measure of effect size defined as the difference in means divided by the standard deviation. The goal of propensity matching methods is to reduce standardized differences to below 0.2, but ideally below 0.1.

### Propensity matching method

We used a propensity matching method to identify non-suspended youth that are similar to suspended youth on the control variables to minimize potential selection bias, using the R MatchIt library [30]: the specific matching method used is called 3:1 exact and nearest-neighbor Mahalanobis matching with replacement, within propensity score calipers of 0.25 standard deviations. The procedure used for matching described in the next two paragraphs will elucidate the meaning of each term in the name of the specific matching method. The matching method identified suspended and non-suspended youth that had similar values of the 67 potential confounders and the estimated propensity score. The estimated propensity score for each participant is the predicted probability that the individual will be suspended: the fitted value of a logistic regression with the outcome of suspension. The predictors in the logistic regression are specified in the below procedure, but it is important to note that the matching procedure can balance on the 67 potential confounders even though only a subset of the 67 variables are included in the propensity score.

The term “3:1 matching” means that we matched 3 non-suspended youth to each suspended youth using the following procedure. The term “exact matching” means that for each suspended youth, exact matching reduced the set of eligible non-suspended youth by requiring that only non-suspended youth with the same daily smoking status and ever-marijuana status could be considered. The term “within propensity score calipers of 0.25 standard deviations” means that we reduced the set of eligible non-suspended youth further to those within 0.25 standard deviations of the estimated propensity score; that is, these non-suspended youth had similar predicted probabilities of suspension. Finally, the term “nearest-neighbor Mahalanobis matching” means that we identified the 3 closest youth according to a correlation-adjusted distance measure of age in years (not rounded) and grade point average; the correlation-adjusted distance measure is named for the statistician Prasanta Chandra Mahalanobis.

We estimated the propensity for each individual to be suspended using a logistic regression with the outcome of a first suspension between 1995 and 96 and predictors of demographic factors (rural residence, Northeast region, lives with both biological parents, male gender, age, born in US, Latino, Asian, and Black race/ethnicity, home language is English); socioeconomic status (SES) (mother high school graduate, mother college graduate, parent is currently employed, per capita household in- come, parent reports enough money to pay bills, father ever in prison [2001]), health and risk behavior factors (experiences with violence, delinquency score, respondent smokes daily, household member smokes, mother smokes, depression score, positive expectancies), educational factors (standardized test score, school attachment, expect to attend college, attend private vs. public school, school is strict on civil order, school is strict on substance use, never truant), and personality factors (parent’s assessment of their child, agreeableness, emotional stability, parental closeness, systematic vs. gut-feeling decision making).

### Statistical analysis within the matched sample

STIs were rare outcomes, so we used logistic regression to predict chlamydia and trichomoniasis in the unmatched and matched samples, controlling for baseline age, race/ethnicity, gender, and household income tertiles [21]. Gonorrhea was rare, with only 21 cases, so we only estimated crude odds ratios, not adjusted odds ratios. Using control variables measured at baseline avoids bias towards the null from using factors that were intermediate between suspension and STIs [31].

We used causal mediation analysis to evaluate whether pre-treatment and post-treatment variables mediated the relationship between suspension and STIs [32]. This study evaluated 34 post-suspension variables for mediation: marriage; educational attainment and predictors of educational attainment (e.g., full-time college attendance, community versus four year college matriculation, enrollment gap prior to matriculation); criminal justice outcomes (ever arrested, convicted as adult, arrested as minor, convicted as minor); sexual orientation (identify as lesbian/gay/bisexual (LGB), publicly open as LGB); employment status (full-time, day shift); substance use (ever smoker, current smoker, binge drinking); sexual risk behavior (partner has STI, frequency of sex in the past year, number of partners in past year, number of partner in lifetime, condom use frequency); personality (impulsivity, self-esteem); and expulsion.

We used sensitivity analysis for multiple controls to assess whether observed differences could be attributed to unobserved variables [33–35].

## Results

### Suspension risks

**Fig. 2:**
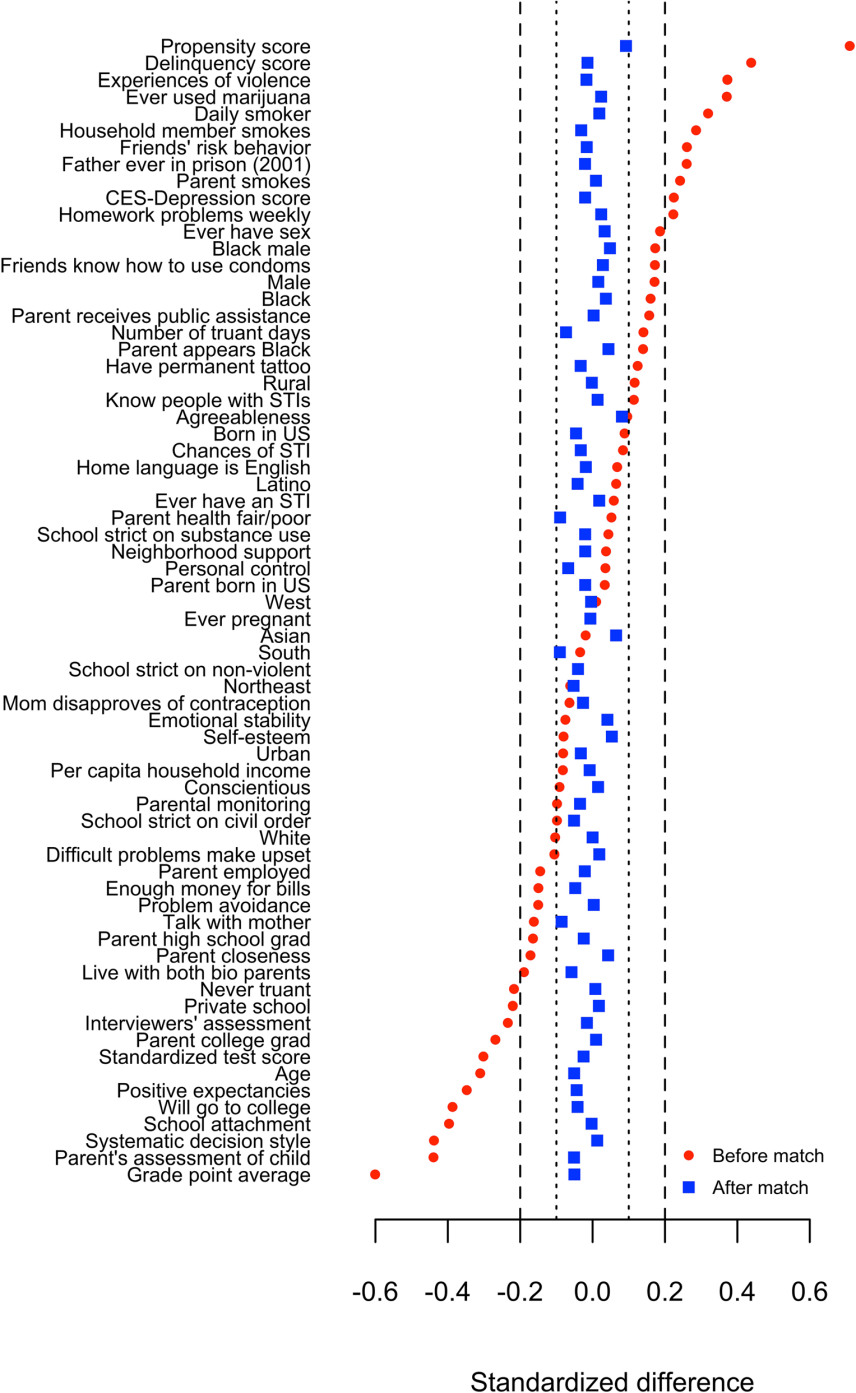
Comparison of standardized differences of baseline factors before and after matching, comparing students who were suspended for the first time between 1995 and 1996 with students who had never been suspended as of 1996

Among 7748 youth with no history of expulsion or suspension, 380 were suspended between 1995 and 1996. The model matched 923 never-suspended youth to the 380 suspended youth and balanced on 67 variables plus the estimated propensity score (Fig. 2).

Risk factors for suspension (associated with greater risk of suspension) included higher delinquency score, more experiences of violence, lifetime marijuana use, daily smoking, household member smoking, friend’s risk behaviors, having a father in prison, parental smoking, and depression score. Protective factors (associated with lower risk of suspension) included never having been truant, attending a private school, interviewer’s positive impressions, parent having graduated college, higher standardized test score, older age, positive expectancies, college intentions, school attachment, systematic decision style, parent’s positive assessment of their child, and higher grades (Fig. 2). After matching, suspended and never-suspended youth had similar values of all 67 factors plus the propensity score (Fig. 2).

### Racial disparities in STIs

Respondents who identified as Black were more likely to test positive for trichomoniasis and chlamydia than respondents who did not identify as Black, both in the full sample and after stratifying on suspension status. In the full sample, 5.4% of Black respondents vs. 1.3% of non-Black respondents tested positive for trichomoniasis (*p* < 0.001); 10.0% vs. 2.7% tested positive for chlamydia (*p* < 0.001) (Table 1A).

**Table 1:** Association between (A) Black identity and STI test results 5 years after suspension, stratified by suspension status; (B) suspension and STI test results 5 years after suspension, stratified by Black identity

A.									
	All			Suspended			Not suspended		
	Black, %	Non-Black, %	P	Black, %	Non-Black, %	P	Black, %	Non-Black, %	P
Trichomoniasis	5.4	1.3	< 0.001	12.1	2.5	0.008	4.8	1.2	0.001
Chlamydia	10.0	2.7	< 0.001	13.2	2.9	0.006	9.7	2.6	0.001
B.									
	Black					Non-Black			
	All, %	Suspended, %		Not suspended, %	P	All, %	Suspended, %	Not suspended, %	P
Trichomoniasis	5.4	12.1		4.8	0.04	1.3	2.5	1.2	0.2
Chlamydia	10.0	13.2		9.7	0.3	2.7	2.9	2.6	0.8

Among Black respondents, 12.1% of suspended young adults tested positive for trichomoniasis compared with 4.8% of non-suspended young adults (*p* = 0.04), but there was no difference for chlamydia (Table 1B). Among non-Black respondents, suspension did not predict a greater risk of testing positive for either trichomoniasis or chlamydia (Table 1B).

### Associations between suspension and STIs

In our sample, 2.00% tested positive for trichomoniasis, which was more common among suspended than non-suspended youth (4.88% vs. 1.85%, *p* < 0.001). Suspended youth had 2.7 times the odds of trichomoniasis than non-suspended youth (Crude OR = 2.72, 95% CI (1.59, 4.38), p < 0.001) The association was attenuated after controlling for race/ethnicity, gender, and income: suspended youth had 2.5 times the odds of testing positive for trichomoniasis (Table 2). After matching, compared with youth who had never been suspended in 1996, youth who were suspended for the first time between 1995 and 1996 had 2.9 times the odds of trichomoniasis, after adjusting for race, gender, and income (Table 2). Before matching, trichomoniasis was more common among black youth, but that association with race was no longer evident in the matched sample (Table 2). The effect is sensitive to a factor of Γ ≤ 1.65.

**Table 2:** Association between a first suspension and positive test for trichomoniasis five years after suspension, within the full sample and matched sample

	Without matching (*n* = 7255)		With matching (*n* = 1227)	
Predictor	OR (95% CI)	P	OR (95% CI)	P
Suspended	2.50 (1.45, 4.10)	< 0.001	2.87 (1.40, 5.99)	< 0.001
Black	2.34 (1.42, 3.95)	< 0.001	1.73 (0.59, 5.71)	0.34
Latino	0.63 (0.33, 1.13)	0.14	0.23 (0.03, 0.99)	0.05
White	0.53 (0.32, 0.89)	0.01	0.38 (0.12, 1.24)	0.10
Male	0.59 (0.40, 0.84)	< 0.001	0.48 (0.22, 1.01)	0.06
Middle household income tertile	0.87 (0.59, 1.27)	0.50	0.38 (0.15, 0.88)	0.03
Top household income tertile	0.64 (0.41, 0.99)	0.05	0.24 (0.05, 0.73)	0.03

Below-median household income explained about 9% of the association between suspension and trichomoniasis (*p* = 0.02). Among pre-suspension variables: race, gender, STI history, sexual debut, mother’s educational attainment, and mother’s disapproval of birth control did not mediate the association between suspension and trichomoniasis. None of the 34 evaluated post-suspension variables mediated the association between suspension and trichomoniasis at the 0.05 level. However, 6% of trichomoniasis was mediated by expulsion (*p* = 0.06) and 4% by having a high school diploma (*p* = 0.14). The pathway between suspension and trichomoniasis was not significantly explained by subsequent criminal justice involvement or lower educational attainment.

In our sample, 3.95% tested positive for chlamydia, which did not differ between suspended and non-suspended youth (5.39% vs. 3.87%, *p* = 0.18). Suspended youth did not differ from non-suspended youth in the adjusted odds of a positive chlamydia test in multivariate regression, both before and after matching (not shown.)

In 2001, 0.30% tested positive for gonorrhea, which was more common among suspended than non-suspended youth (0.88% vs. 0.27%, *p* = 0.05). Suspended youth had greater odds of gonorrhea than non-suspended youth (Crude OR = 3.23 (0.95, 11.03), *p* = 0.06). The sample had only 21 cases of gonorrhea, which did not permit further analysis.

Impulsivity didn’t differ between suspended and matched non-suspended youth in either unadjusted or adjusted models: on average, suspended youth were 0.009 points more impulsive than matched non-suspended youths in multivariate linear regression (*p* = 0.54).

## Discussion

Temporarily removing students from school for the first time predicted greater risk of testing positive for trichomoniasis and gonorrhea 5 years later, and the association between suspension and trichomoniasis was greater among low-income youth. Out-of-school suspension is common and promoted by federal zero-tolerance policy, with over a third of youth suspended over a K–12 school career [5], suggesting a large proportion of youth who are at greater than average STI risk. Suspended youth are at greater risk of arrest [16] and arrested youth have greater STI risk [36], but this analysis suggests that increased STI risk exists even among those who are suspended but not subsequently arrested.

The association between suspension and trichomoniasis could be explained by the theory of labeling and deviance amplification: youth who are suspended may be labeled as deviant and associate with more deviant peers and riskier sexual networks [37]. Past research suggests that police stops and first arrests predict subsequent arrests, and school suspension predicts arrests, which have been explained as effects of labeling and secondary deviance [10, 11, 16].

Out-of-school suspension separates youth from school environments where they have positive influences, including access to lower risk sexual networks. School discipline policies that maintain greater school attachment for all students may reduce students’ propensity to become involved with high-risk sexual networks and decrease their STI risk.

Youth from households with below-median income were more vulnerable to the effects of suspension. Youth from above-median income households have access to social capital resources for overcoming setbacks, including being more likely to come from two-parent households and having college-educated parents who know how to navigate educational systems and mediate with schools [38]. School environments may also be more forgiving of deviation among higher income youth. Youth from below-median income households rely more on school environments for obtaining social capital; when these youth were excluded from school, they were more likely to be left to navigate the situation themselves, which may be because parents with below-median income may have less knowledge or time to assist their children in seeking second chances [38].

Trichomoniasis, caused by the parasite *T. vaginalis*, is the most common curable STI in the US, with prevalence at the time of this study of 3.1% among all women and 13.3% among non-Hispanic Black women, of whom 85% reported no symptoms [39]. Trichomoniasis appears to increase the risk of HIV infection [40], in women is associated with adverse birth outcomes [41], and in men may be associated with subfertility or infertility [42].

Trichomoniasis is more common in women over 35 than younger women [40]. However, this study finds substantial trichomoniasis prevalence among Black and low-income young adults ages 18–25 who were suspended, suggesting trichomoniasis as a target of STI/HIV prevention interventions in vulnerable populations of young adults. STI/HIV prevention interventions in young adults often measure criminal justice system involvement; this study suggests that interventions that measure suspension and expulsion could measure STI risk in a population at higher risk of criminal justice involvement (13.)

Public health workers reach adolescents through their schools for essential health services, such as health screenings and school-based clinics. STI prevention in schools emphasizes testing, treating, and offering condoms within the schools. This research suggests that schools may also impact the health of adolescents through their disciplinary policies. Previous researchers have called for public health to help to improve school climate, including reducing harsh school discipline policy [2].

### Strengths and limitations

This study uses STI tests rather than self-reported STIs, which minimizes both under-report of STIs and self-report bias. STIs are under-reported because many cases of chlamydia and about 80% of trichomoniasis cases are asymptomatic in both men and women [40]. Suspended youth may differ in their likelihood of taking STI tests or reporting STI diagnoses, leading self-reported STIs to have differential misreporting.

It’s unknown why trichomoniasis and gonorrhea were associated with suspension, but chlamydia was not. However, many STI prevention interventions reduce chlamydia but do not reduce gonorrhea or chlamydia. In this case, trichomonas was screened more accurately in this study than in clinical settings. As noted earlier, this study’s trichomoniasis test had sensitivity of 91% in women and 89% in men, which is much greater than the 50% sensitivity of the standard wet-mount test that was the only FDA-approved method until recently, so participants recently screened for trichomoniasis would not have been treated in clinical settings. However, the 80% rate of asymptomatic trichomonas infections suggests many participants would not have seen by clinicians or tested [39].

Outcomes were measured 5 years after suspension and the matched sample comes from a nationally representative sample; previous studies of health sequelae of suspension followed youth only for one year and only in Washington State and Australia [12–14]. However, these findings may not generalize to current adolescents. Since this data was gathered, the federal government promoted alternatives to suspension during the years 2011–16, and isolated states and school districts continue attempts to reduce suspension, although suspension continues to be widespread [25]. Public health recognizes that education influences adolescent health [2, 43], but Add Health remains the only national dataset that both measures school suspension and tests youth for STIs.

This study may underestimate the association between suspension and trichomoniasis. Trichomoniasis testing methods have become more sensitive since these results were obtained in 2001 [44]. Low sensitivity biases estimates of association towards the null because the misclassification is not differential by suspension status [45–47], suggesting that if this study could be repeated using current, more sensitive trichomoniasis testing, the association between suspension and STI would be stronger.

This study uses matched sampling, which identifies non-suspended youth who are similar to suspended youth on 67 factors, which minimizes potential for confounding on pre-suspension factors. Matching on potential confounders reduces the possibility that extraneous factors explain the observed associations; for example, disadvantaged youth are both more likely to be suspended and acquire STIs [21]. This study also used first suspensions in a subsample of students who reported no previous expulsions or suspensions at baseline; suspended and non-suspended youth were matched on pre-suspension factors, so that the temporal ordering of potential confounders and suspension is clear.

## Conclusions

School suspension is associated with greater risk of testing positive for trichomoniasis five years after suspension, suggesting that behavioral and social network changes by suspended youth may persist and result in lasting health effects. Clinicians can use history of school suspension and expulsion as markers of health risks.

## Data Availability

The Add Health restricted data is available by application through the Add Health website (http://www.cpc.unc.edu/addhealth)

## Appendix

### Description of control variables

The control variables were 67 potential confounders of the relationship between suspension and STIs, derived from 158 survey items. The 67 control variables are listed in the 9 categories for ease of reading, but these categories do not have statistical implications: demographics, socioeconomic status, sexual risk-taking, relationships with adults, educational factors, parents’ risk behavior, substance use, personality and mental health, and deviance.

**Demographics** (16 variables from 10 items): Demographics includes gender; nativity; parent’s nativity; age in years; Latino ethnicity, White, Asian, and Black race; male-Black interaction term; respondent’s parent apparent race (Black vs. not); whether the respondents primary home language is English; region of country; and urban/suburban/rural residence.

**Socioeconomic status** (SES) (6 variables from 6 items): Socioeconomic status (SES) is associated with both suspension and STIs: parent graduated high school; parent graduated 4-year college; parent- reported household income divided by the square root of the household size [31]; parent reported having enough money to pay bills; parent receives public assistance; and parent is currently employed.

**Sexual risk-taking** (7 variables from 7 items): may be associated with suspension and is associated with STIs: ever having had any of 10 sexually transmitted infections (STI); having ever been pregnant; having ever had sexual intercourse; perceived chances of STIs; number of people they know who have had an STI; friends’ knowledge about condoms; and mother’s attitude towards contraception.

**Relationships with adults** (6 variables from 34 items): Adolescents’ relationships with their parents and adults in their neighborhood predict likelihood of suspension and sexual risk-taking: lives with both biological parents; parents assessment of relationship with child (how is child’s life going, get along with child, trust child, child doesn’t have a bad temper, 4 items, alpha = 0.67); parental closeness (aggregate of 14 items such as perceived love and warmth, satisfaction with relationship, alpha = 0.81); talk with mother (talk with mother about social, personal, school issues, 4 items, alpha = 0.62); parental monitoring (parents let respondent make own decisions about weekday bedtime, weekend curfew, how much TV, 7 items, alpha = 0.70); and neighborhood support (e.g., know most of the people in the neighborhood, average of 4 binary items, alpha = 0.72).

**Personal and school-level educational factors** (12 variables from 37 items): Educational factors predict educational attainment and could predict school suspension: standardized test score (Add Health Peabody Vocabulary Test); expectations to attend college; whether the respondent attends a private school; grade point average (average of 4 self-reported grades, alpha = 0.72); positive expectations for the future (aggregate variable of 5 items such as will not be killed by age 21, will live to age 35, alpha = 0.61); school attachment (aggregate variable of 9 items including feeling safe at school, problems with teachers, problems completing homework, alpha = 0.78); school is strict on substance use (top quartile of administrator-reported school discipline policy for alcohol, drugs, and smoking, 8 items, alpha = 0.97); school is strict on civil order (top quartile of administrator-reported school discipline policy for offenses such as stealing school property and verbally abusing a teacher, 7 items, alpha = 0.73); school is strict on non-violent offenses; number of truant days; and having never been truant.

**Parent risk-taking** (4 variables from 4 items): Youth with parents with greater health risks may have greater likelihood of risk behavior that could lead to both suspension and STIs: parent-reported parent smoking; household member smokes; parent’s health is fair or poor; and one item from 2001: whether the respondents father was ever in prison.

**Respondent’s and friends’ substance use** (3 variables from 5 items): Substance use could lead to both suspension and is associated with STIs: lifetime marijuana use, regular smoking status, and friends’ substance use (number of friends who drink alcohol monthly, use marijuana monthly, smoke daily, 3 items, alpha = 0.72).

**Personality and mental health** (9 variables from 28 items): Personality and mental health predicts both deviance and others’ response to an individual’s deviant behavior: self-esteem (aggregate of 11 factors modified from Rosenberg’s scale, alpha = 0.88); conscientiousness (aggregate of 5 items describing systematic approach to solving problems, alpha = 0.78); systematic versus gut-feeling decision-making was measured by the Likert item, “When making decisions, you usually go with your gut feeling without thinking too much about the consequences of each alternative.” where higher means more systematic decision-making style, emotional stability (aggregate of 6 items including have a lot of good qualities, a lot to be proud of, alpha = 0.87) [48]; agreeableness (sum of 3 items: never argue with anyone, never get sad, never criticize other people, alpha = 0.63); personal control was measured by the Likert-scale item “When you get what you want, its usually because you worked hard for it.”; problem avoidance was measured by the Likert-scale items “You usually go out of your way to avoid having to deal with problems in your life.” and “Difficult problems make you very upset.”; and depression was measured by the modified Center for Epidemiologic Studies Depression screen score (19 items, alpha = 0.86).

**Deviance** (4 variables from 27 items): Deviance predicts both likelihood of suspension and STIs: delinquency was the sum of 15 binary items including running away, hurting someone so badly that they needed medical care, participating in a group fight, lying to parents, and stealing <$50 and > $50 (alpha = 0.80); experiences with violence was the sum of 8 binary variables, such as saw shooting, was shot, shot another (alpha = 0.75.); having a permanent tattoo; and the interviewers assessment of students appearance (attractive, personality attractive, well-groomed, 3 items, alpha = 0.74).

## Abbreviations

FDA: United States Food and Drug administration
HIV: Human immunodeficiency virus
SES: Socioeconomic status
STI: Sexually transmitted infection
